# Supplementary material for: Matrix Metalloproteinases Expression Is Associated with SARS-CoV-2-Induced Lung Pathology and Extracellular-Matrix Remodeling in K18-hACE2 Mice
Source: Viruses. 2022 Jul 26;14(8):1627. doi: 10.3390/v14081627 (PMC9332556; doi:10.3390/v14081627)
Supplement: Supplementary file 1 [file viruses-14-01627-s001.zip › viruses-1722401-SI.pdf]

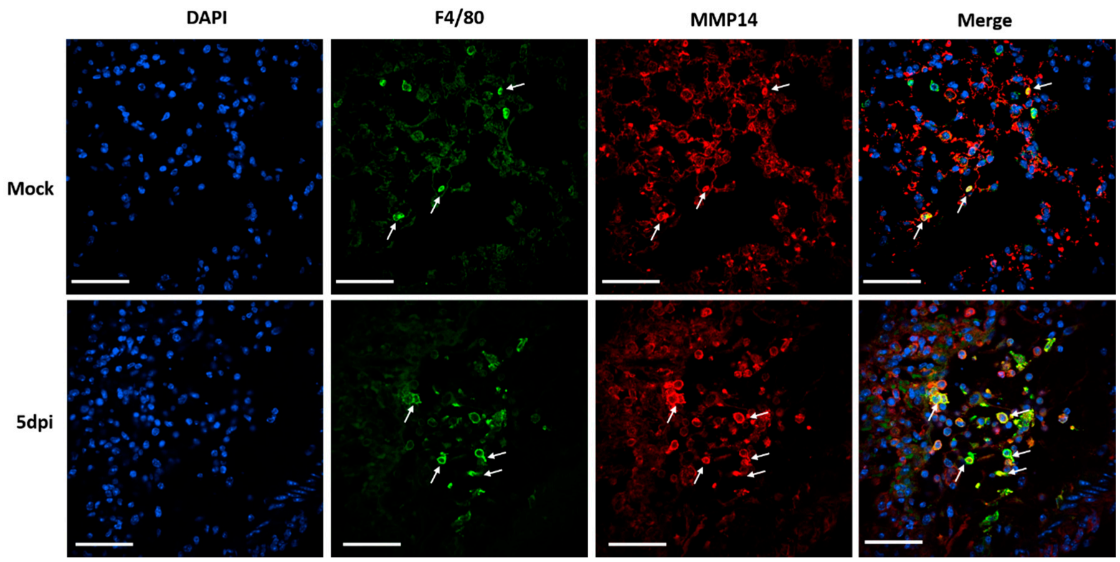

Supplement Figure S1. MMP14 expression by alveolar macrophages in the lungs of K18-hACE2 mice after SARS-CoV-2 infection

Representative images of lung sections labeled for F4/80 marker for macrophages (green) and MMP14 (red) indicating co-localization. Arrowheads point to MMP14 or F4/80 stained cells and to their co-localization. Scale bar: 50 µm.

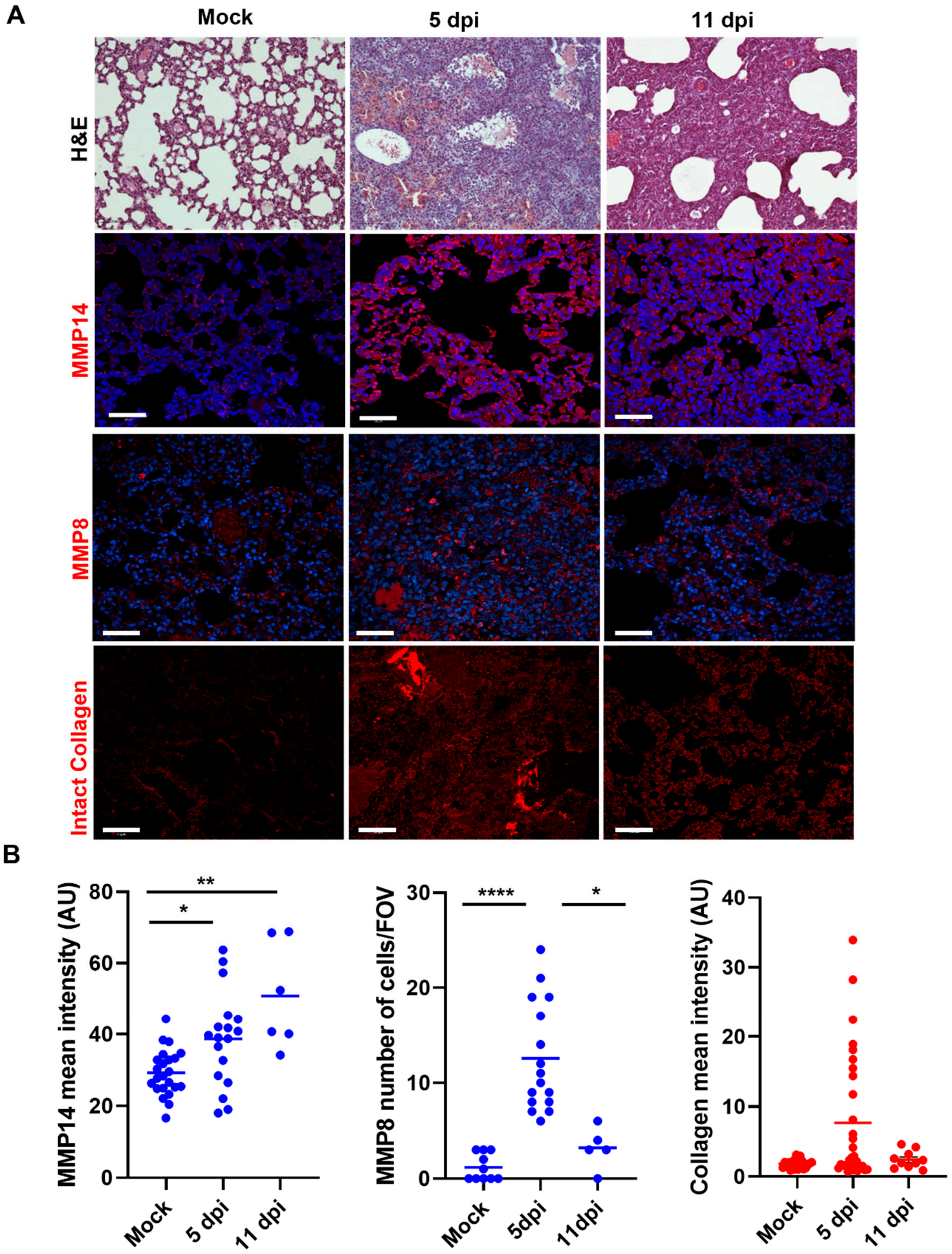

Supplement Figure S2. MMP14 and MMP8 expression and collagen degradation in the lung of COVID-19 hamster model

(A) Representative images of lung sections labeled for H&E (upper panel), MMP14 and MMP8 (middle panel) and Collagen (CHP) (lower panel) followed by quantitative analysis (B) at 5- and 11-

days post SARS-CoV-2 infection compared to mock. Scale bar: 50 µm. For each group n=3-5 hamsters. Inner lines represent the median. Statistical significance was calculated using Kruskal-Wallis nonparametric test $*p<0.05$, $**p<0.005$, $****p<0.0001$
